# Influence of limb dominance on body and jump asymmetries in elite female handball

**DOI:** 10.1038/s41598-023-46615-w

**Published:** 2023-11-07

**Authors:** Laura Carrasco-Fernández, Manuel García-Sillero, Jose Manuel Jurado-Castro, Dasiel Oscar Borroto-Escuela, Jerónimo García-Romero, Javier Benítez-Porres

**Affiliations:** 1 Department of Human Physiology, Physical Education and Sport, Faculty of Medicine, University of Málaga, Bulevar Louis Pasteur, 25, 29010 Malaga, Spain; 2 Metabolism and Investigation Unit, Maimonides Biomedical Research Institute of Cordoba (IMIBIC), Reina Sofia University Hospital, University of Cordoba, 14004 Córdoba, Spain; 3 CIBERobn Physiopathology of Obesity and Nutrition, Centre of Biomedical Research Network, Institute of Health Carlos III, 28029 Madrid, Spain; 4 Escuela Universitaria de Osuna, Teaching Center Attached to the University of Seville, 41640 Seville, Spain; 5 Department of Neuroscience, Karolinska Institutet, Stockholm, Sweden

**Keywords:** Physiology, Musculoskeletal system

## Abstract

Handball is a team sport subjected to asymmetric actions that require high physical capacity demands on players. The development of large asymmetries could negatively affect sports performance. However, few studies have analyzed body composition and the force asymmetries in elite female handball players. The aim of this study was to analyze the presence of asymmetries based on limb dominance in body composition parameters and lower limb power in jumping performances in an elite women’s handball team. An entire elite women’s handball team, comprised by of 14 players, was analyzed. Dual X-ray Absorptiometry (DXA) and bioimpedance were used to analyze body composition. Force plates were used to evaluate jump performance. Results show the presence of differences between all the players in the different parameters of the CMJ jump. In addition, an asymmetry between the power of the dominant and non-dominant lower limb was observed between the players. The results show differences in muscle mass between the upper limbs, but not in the lower limbs in terms of both muscle and fat mass. However, there were no crossed asymmetries or significant differences between members based on dominance. The results suggest that the presence of asymmetries does not have to be one of the main parameters to be taken into account by coaches in elite athletes and to highlight the importance of including specific analyzes of body composition and sports performance in an individualized way.

## Introduction

Handball is characterized as a team sport that involves high-intensity asymmetric actions, requiring significant physical demands on the player^1^. It is a contact sport that includes physical efforts such as running, jumping, ball throwing, various movements, turns, and changes in speed and directions^2^.

As the first part of the kinetic chain, the contralateral lower extremity plays a critical role in maintaining the dynamics of balance and initiating the forces transferred through the trunk during ball throwing^3^. However, the repeated unilateral loads that occur in handball can lead to crossed asymmetries in athletes, which can increase imbalances between the extremities^4^.

Several studies have explored the relationship between asymmetries and sports performance, observing that a high imbalance between the dominant and non-dominant limbs can affect sports performance variables such as speed and strength^5,6^**.** Previous studies have indicated the importance of studying parameters such as the concentric and eccentric Rate of Force Development (RFD) and reactive strength index (RSI) for both improving sports performance and preventing sports injuries. This is due to its direct and indirect relationship with the elastic energy of the muscle–tendon system^7^.

In this sense, the presence of large asymmetries could negatively affect sports performance through vertical and horizontal jump, considering the presence of asymmetries from 6 to 7% in horizontal jump tests and an increase in asymmetries of 10% in unilateral vertical jump tests^4^. However, an asymmetry value of less than 7% between the dominant and non-dominant limb is considered small and does not demonstrate a significant relationship with sports performance^8^. It should be noted that depending on the test performed, the asymmetry values between limbs can be different. In addition, the asymmetries do not depend only on the type of sport, but also on the individual demand on each player on the court^9,10^.

Nevertheless, limb asymmetries have also been related as an important risk factor in the incidence of sports injuries^8^**.** As a consequence, there are a large number of studies that focus on the evaluation of segmental body composition and anthropometric factors in elite sports, considering them essential variables in the context of sports performance and injury prevention^11^.

Kale and Akdoğan^12^, indicate the importance of assessing body composition and anthropometric variations throughout a competition cycle in female handball players. In this way, it is intended to prevent sudden changes during the season that may impair sports performance, or even reinstatement of the players after a period of flack of training due to injuries^12^. In this sense, it is essential to remember that achieving optimal body composition values can contribute to reducing the risk of asymmetries and injuries and facilitate the rehabilitation process for a return to play^13^.

One of the most widely used body composition assessment techniques in sport is bioelectrical impedance^14,15^, which shows a high level of reliability with the gold standard in this field, such as dual energy X-ray absorptiometry or DEXA^16^. Among the variables obtained by the Z, is the phase angle, which is obtained from the relationship between the measurements of resistance (R) and reactance (Xc). This index being equal to the arctangent of the reactance divided by the resistance^17^. Scientific evidence reflects that a decrease in the phase angle is associated with the presence of impaired integrity and injury to the cell membrane, which leads to a greater risk of acute injury or damage due to tissue over load and decreased the sport performance^18^**.**

However, few studies have analyzed body composition asymmetries and rate off force development taking into account limb dominance in elite female handball players and the relationship to athletic performance^9,19^. For this reason, the main objective of this study was to assess whether there are asymmetries bases on the dominance of the limbs between players. A secondary objective was to analyze whether the presence of asymmetries in body composition parameters between lower extremities showed a relationship with the development of asymmetries in muscle power parameters during jumping performance. To achieve this, the study was included segmental analyses, focusing primarily on body composition variables and the countermovement jump (CMJ), enabling to evaluate the presence of asymmetries between dominant and non-dominant limbs.

In relation to the proposed objectives, it was hypothesized that the presence of slight asymmetries will be visible in most players as it is a sport characterized by asymmetric actions, with the dominant limb being the one that presents the most optimal values of body composition and strength and influencing the jump performance.

## Materials and methods

### Study design

In this research, a cross-sectional experimental design was carried out, where participants were selected intentionally, non-probabilistically and for convenience. The female handball players participated of your own accord and the data collection presented a duration of eight weeks, within which two previous weeks of familiarization were included.

### Population

A total of 14 elite female handball players participated (Aged: 27 ± 4.96, height: 170.11 ± 7.7 cm, BMI: 22.9 ± 1.7 kg m^2^). The players were recruited from the Málaga Costa del Sol Women’s Handball Club, current European champions (EHF European Cup, 2021).

Regarding the inclusion criteria, the players of the team were selected taking into account the position of each player, the lower and upper dominant limb, showing that the dominant upper limb in throwing the ball was the contralateral in relation to the lower limb dominant during the jump. In addition, the presence or history of injuries related to sports practice during their professional career in handball. No one of those players presented an injury at the time of data collection or had suffered a significant injury in the last 6 months that could interfere with jump performance. However, two players were excluded from the jump test due to the presence of injuries. Furthermore, the menstrual cycle of the players was not taken into account at the time of data collection.

### Process

The technical team of the Costa del Sol Women’s Handball Club (Málaga) team was contacted and they were informed of the entire procedure that would be carried out. The search protocol was carried out in accordance with the ethical guidelines of the Declaration of Helsinki^20^ and was approved by the ethics committee at University of Málaga (code: 38-2019-H). The subjects were informed of the conditions of the study and were aware of the possible risks of the experiment and signed an informed consent ledging their willingness to participate.

**Figure 1:**
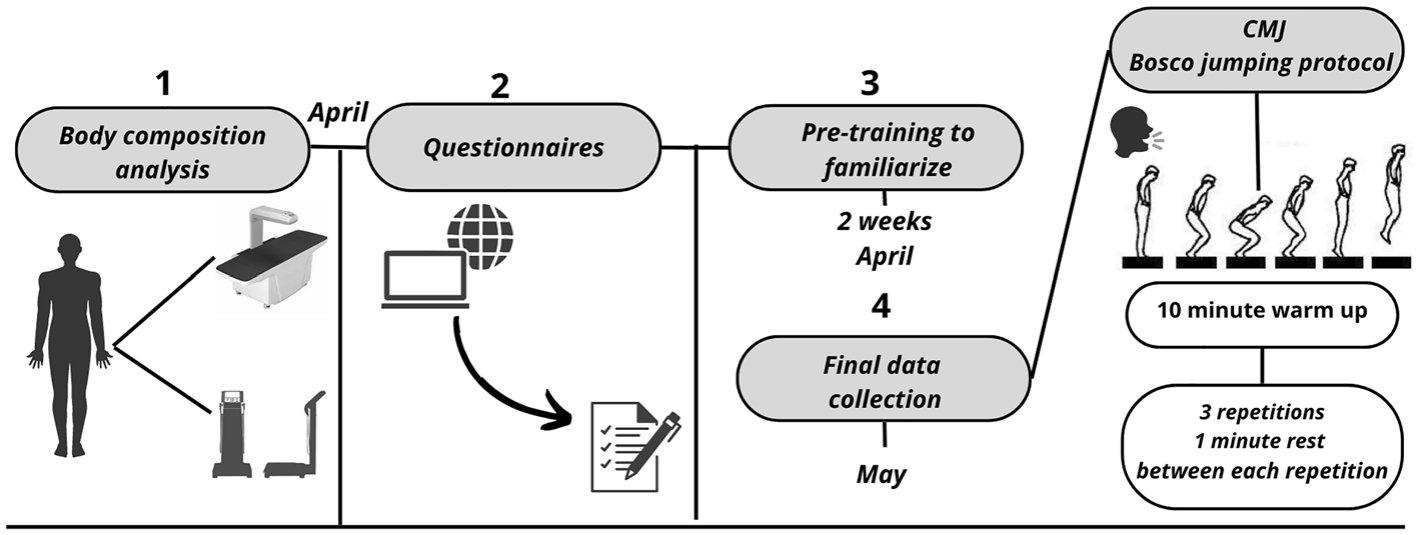
Organization of data collection.

All measurements and tests were carried out in the laboratory of effort physiology and sport medicine at the University of Malaga, in the Carranque sports center and in Eshmún Sport Clinic in Malaga (Fig. 1).

### Data collection techniques and instruments

#### Body composition

For the analysis of body composition, body weight was previously measured with a scale (SECA, Hamburg) and body height using a wall-mounted stadiometer (Holtain Ltd, United Kingdom).

Subsequently, a bone densitometry was consecutively performed on each subject using dual X-ray absorptiometry using the bone densitometer-DXA (APEX software version 5.6.0.7, Hologic Horizon A, USA)^16,21^.

DXA was calibrated with phantoms according to the manufacturer’s guidelines each day before measurement. It was indicated in each exploration that the participants had to come fasting and not have trained the day before the body composition analysis. In addition, the participants wore light clothing and were asked to remove any materials that might attenuate the X-ray beam. DXA provided reproducible estimates of total and segmental distribution parameters of body composition including body fat percentage, body fat mass, total lean body mass, total body mass, leg fat mass, torso fat percentage and torso fat mass among others.

Moreover, segmental multifrequency data were obtained that accurately determined total, intracellular and extracellular body water, impedance (Xc and R) and phase angle (Z) in the five body segments (right arm, left arm, trunk, right leg, left leg) by bioimpedance (BIA)^15,22^ using the Inbody® 770 model (Inbody 770, Inbody, Seoul, Korea).

#### Bilateral jump performance

As for the criterion device for evaluating jump performance, the force plates Force Decks Dual Plate System (Vald Performance, Albion, Australia) was used^23^.

For the evaluation of jumping performance, the Bosco jumping protocol was followed for performing the bilateral CMJ^24^. In addition, *Vald Performance* makes it possible to know the level of asymmetry between the right and left leg, taking into account the dominant lower limb in the jump test of each player. This allows knowing unilateral values of the dominant and non-dominant lower limb despite using the bilateral CMJ test^25^.

All subjects underwent a two-week pre-training to familiarize themselves with the protocol that was incorporated during general team training. Each subject, after performing a previous warm-up, performed a total of three repetitions in a row with a 1 min rest between tests and the mean of the three repetitions was selected in the statistical analysis^26^. In addition, a standardized verbal stimulus was given to each participant during the test in order to achieve maximum performance in the jump.

Performance variables such as jump height, RFD, RSI, kinetic parameters such as average, maximum or impulse force and kinematics, highlighting the average flight time, are selected^25^. Furthermore, the analysis of the asymmetry values of these parameters between the dominant and non-dominant limb during the different phases of the jump was carried out; concentric and eccentric phase, acceleration and deceleration phase, and takeoff and landing phase.

### Statistical analyses

The values presented in the descriptive tables show the mean and standard deviation. To determine the normality of the variables, Shapiro–Wilk tests were conducted, and Levene’s test was used to test for variance equality. A one-sample T-test was performed to compare differences in CMJ performance and asymmetry among the players. This analysis shows the results with percentage values and not with p-values. A paired-samples t-test was conducted to analyze bilateral variability in body composition and dominant and non-dominant limbs. Linear regression was used to detect possible relationships between the asymmetries in mass between the dominant and non-dominant limbs and the presence of asymmetries in jump parameters. All regression models were assessed by model checking, including investigating the linearity of effects on outcomes, ensuring normal distribution, and homogeneous variance. Statistical significance was set at *p* < 0.05, and all statistical procedures were performed using SPSS version 25 (SPSS Inc., Chicago, USA).

## Results

Descriptive statistics for physical parameters and total body composition are presented in Table 1. In 12 participants, the dominant jumping leg was the left, while only two had the right as their dominant jumping leg.

**Table 1:** Descriptive statistics of physical parameters and total body composition.

Parameters	Mean ± SD
Age (years)	27.6 ± 4.9
Weight (kg)	67.3 ± 8.8
Height (cm)	170.1 ± 7.7
Total body fat (kg)	16.8 ± 4
Total lean body mass (kg)	50.5 ± 5.2
Total body fat (%)	24.7 ± 2.9
Visceral abdominal body fat (%)	22.8 ± 3.44
Total body water (L)	39.3 ± 4.6
Extracelular body water (L)	24.7 ± 2.8
Intracelular body water (L)	14.6 ± 1.75
Full body phase angle 50 kHz (º)	6.3 ± 0.4

*Note:* Data is shown as mean and standard deviation (SD).

All players presented differences in the different parameters of the CMJ jump between them. Additionally, a percentage of asymmetry was observed between the left and right lower limb power among the players, mainly in the eccentric and in the maximal landing phase (Table 2).

**Table 2:** Descriptive CMJ parameters and asymmetry between left and right leg power in lower limb among female players.

Parameters	Mean ± SD
Jump height (cm)	29.1 ± 4.5
Maximal concentric velocity (m/s)	2.5 ± 0.2
Mean concentric force (w)	1237.5 ± 173.1
Maximal eccentric force (w)	1369.2 ± 252
Mean eccentric force (w)	666 ± 98
Maximal takeoff force (w)	1519.2 ± 235
Asymmetry between left and right leg (%)	
Concentric force D/Non-D (%)	7.3 ± 5.6
Eccentric force D/Non-D (%)	7.6 ± 5.4
RFD of eccentric deceleration D/Non-D (%)	17.9 ± 11.7
Maximal takeoff force D/Non-D (%)	5.8 ± 5.3
Maximal landing force D/Non-D (%)	14.2 ± 8.9

*Note:* Data is shown as mean and standard deviation (SD). Asymmetry variables as represented as a percentage (%) D, dominant leg; Non-D, non-dominant leg; RFD, rate of force development; w, watts.

The results of the bilateral variability analysis are presented in Table 3, which showed that the lean mass of the dominant upper extremity is significantly greater than that of the non-dominant side. However, no significant difference was found in fat mass between the upper limbs. The lower limbs, despite the fact that 85.7% (n = 12) of the players had the left leg as their dominant lower limb, it is reflected in the variability analysis that the lean mass of the right leg is significantly larger compared to the left leg. However, no differences (*p* > 0.05) were found between limbs in the values of total body water (TBW), intracellular body water (IBW), extracellular body water (EBW), electrical impedance and phase angle.

**Table 3:** Differences between dominant vs non-dominant upper and lower extremities, in the variables of lean and fat mass, TBW, ICW, ECW, impedance (50 kHz) and phase angle.

Bilateral variability D/Non-D	Mean ± SD	p-value
Arm lean mass (kg)	0.14 ± 0.17	0.007*
Leg lean mass (kg)	0.14 ± 0.22	0.041*
Arm fat mass (kg)	0.17 ± 0.05	0.181
Leg fat mass (kg)	0.14 ± 0.16	0.006*
Arm TBW (L)	0.02 ± 0.08	0.398
Leg TBW (L)	0.01 ± 0.09	0.590
Arm IBW (L)	0.01 ± 0.05	0.405
Leg IBW (L)	0.02 ± 0.05	0.131
Arm EBW (L)	0.01 ± 0.03	0.393
Leg EBW (L)	0.01 ± 0.04	0.338
Arm impedance (kHz)	5.2 ± 14	0.191
Leg impedance (kHz)	0.5 ± 6.5	0.776
Arm phas eangle (kHz)	0.2 ± 0.3	0.054
Leg phase angle (kHz)	0.1 ± 0.2	0.073

Note: *p < 0.05. Data is shown as mean and standard deviation (SD) of dominant and non-dominant limbs, respectively. D, dominant leg; EBW, extracellular body water; IBW, intracellular body water; Non-D, non-dominant leg; TBW, total body water.

**Figure 2:**
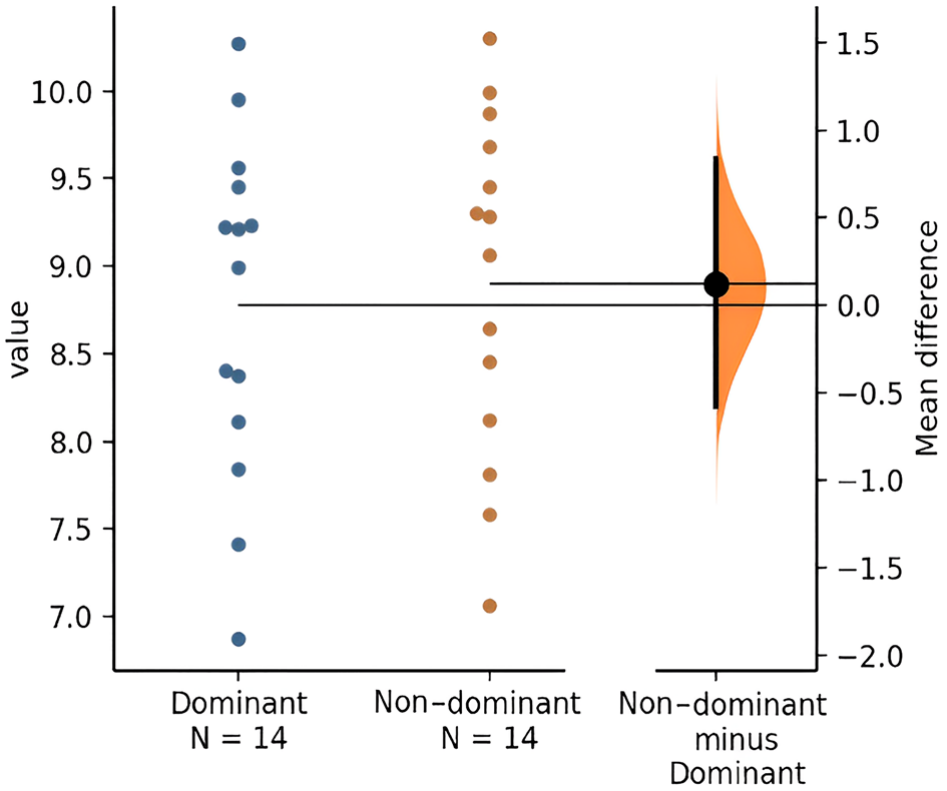
Lean mass (kg) dominant leg vs non-dominant leg during the jump.

The participants did not show differences in lean mass between dominant *vs* non-dominant leg (8.78 ± 0.98 kg *vs* 8.90 ± 0.98 kg; *p* = 0.072) (Fig. 2). There were no changes in lean mass between dominant and non-dominant arm (2.72 ± 0.33 kg *vs* 2.62 ± 0.34; *p* = 0.101).

No associations were observed between the difference in lean mass between dominant and non-dominant leg and the asymmetry between legs in the jump (Table 4).

**Table 4:** Association between the difference in lean mass between dominant and non-dominant leg and the asymmetry between legs in the jump.

*Asymmetry between* dominant and non-dominant leg *(%)*	Difference in lean mass between dominant and non-dominant leg (0.13 ± 0.24 kg)		
	β	p-Value	R^2^
Concentric force D/Non-D (%)	− 0.001	0.965	0.001
Eccentric force D/Non-D (%)	0.020	0.132	0.194
RFD of eccentric deceleration D/Non-D (%)	0.002	0.787	0.007
Maximal take off force D/Non-D (%)	0.001	0.948	0.001
Maximal landing force D/Non-D (%)	0.009	0.244	0.121

D, dominant leg; Non-D, non-dominant leg.

## Discussion

The aim of this research was to analyze the total and segmental body composition, and the power parameters during the jumping performance of an elite women's handball team in order to value the differences between extremities taking into account the dominance of the upper and lower limb. In addition, analyze whether the presence of asymmetries in body composition parameters between lower extremities showed a relationship with the development of asymmetries in muscle power parameters. Regarding the results presented, as in previous studies, in this sample it is observed that the muscle mass of the right upper and lower extremities is significantly higher compared to the left side. In the case of total body fat mass, greater significant differences were also found in the right lower limb in relation to the left side (Table 3)^2,27^. In the study by Lijewski et al.^28^, it was observed significant differences in muscle mass in the right and left limbs, presenting greater muscle mass in the upper and lower right limbs, and Arboix-Alió et al.^29^, found significant differences between the higher performing limb (HPL) and the lower performing limb (LPL).

However, according to Fort-Vanmeerhaeghe et al.^30^, most team sports are typically associated with the development of functional asymmetries, as one limb is used more frequently than the other, leading to the dominant limb having greater strength capacity and better coordination than the non-dominant limb. Furthermore, Van Melick et al.^31^, indicate that the dominant lower limb will be the same in tasks of bilateral mobilization and unilateral stabilization, as in this case the unilateral jump in 70% of women. Nevertheless, in this sample it was observed that despite the fact that most players presented the left limb as the dominant lower limb during the jump test, taking dominance into account. The body composition parameters did not show statistically significant differences between the dominant and non-dominant limbs. In addition, no statistically significant differences were found between limbs in the values of TBW, IBW, EBW, electrical impedance and phase angle (Fig. 2).

In contrast, previous studies have shown that in asymmetric sports such as handball, the contralateral leg to the dominant upper extremity compensates for the weight of the upper limb that throws the ball, leading to the development of crossed asymmetry in the athlete^32^. Furthermore, in this case, there were no crossed asymmetries, which could be justified with the characteristics of each player on the court, highlighting that depending on the position, not all players present unilateral dominance in their actions^33,34^.

Furthermore, previous research has also shown that asymmetry of lean mass, which includes not only muscle mass but also bone mass, can also explain the asymmetry of force and power generated during jumping^35^. However, just as in this study, there are other investigations that have not been able to justify the relationship between lean mass and asymmetries parameters when designing specific training sessions^36^. In this case, it was not observed that the presence of lean mass between the right and left leg showed a significant relationship with the presence of asymmetries in the force parameters during the different phases of the jump (Table 4).

On the other hand, all players showed differences in the CMJ parameters between them. In addition, asymmetries were observed between the dominant and non-dominant leg in the muscle power parameters in the different phases of the jump, mainly highlighting the presence of asymmetries in the eccentric phase and in the landing phase (Table 2).

Barrera- Domínguez et al.^4^, shows the presence of asymmetries greater from 6 to 7% between dominant and non-dominant limb in jump tests is relevant and it increase the risk of development sport injuries and decrease the sport performance. Previous studies have shown that eccentric training has gained more attention, showing more favorable results compared to concentric training, highlighting greater muscle activation, greater lower extremity stiffness, and better athletic performance^10,37^. In addition, various studies have shown that asymmetry values can vary depending on the test used to quantify the imbalance^9,10,38,39^. Madruga-Parera et al.^19^, in their study show that jump tests can be more sensitive for the analysis of asymmetries compared to other tests such as the sprint test, strength tests or the limb symmetry index. It is important to highlight the large number of eccentric actions of the lower limb at high speed that occur in general in team sports, and specifically in handball. The data from our study (17,9% asymmetry RFD of eccentric deceleration) showed a different porcentage in this parameter, which should be taken into account by physical trainers, with a view to improving injury prevention.

On the other hand, this supports the theory of other investigations which have shown that the presence of asymmetries will be specific to each test and therefore it is essential to include different tests during training, highlighting different bilateral and unilateral tests, highlighting the latter by showing better results related to imbalances between limbs^10^. This type of test should not be replaced, given its validity, but perhaps complemented with other more specific tests, as recent research has shown^40^.

However, it is important to highlight the importance of including more specific exercises in different planes of movement such as unilateral plyometric training and variables aimed at improving the execution of specific handball tasks, noting that they can be more useful to detect the presence of asymmetries between extremities^10,37^.

By contrast, Bishop et al.^10^ showed that the values of the asymmetries of the individual players were very different compared to the results based on the average values of all the metrics. For this reason, the importance of including an analysis of body composition during the different periods of the season is indicated to avoid imbalances in sports performance. In addition, to evaluate more specific parameters such as the rate of force development and reactive force to improve sports performance and reduce the development of asymmetries through specific training programs^41^.

However, this study does not show that dominance between limbs and the assessment of asymmetries in a generic test such as the CMJ is a relevant method for current training programs for elite handball teams. For this reason, it highlights the importance of assessing the players individually, including more specific exercises, highlighting the importance of eccentric actions, including the mechanics of changing direction, running, jumping and landing. or even the rapid stretch shortening cycle demands that occur in elite women's handball^42^.

The main limitation of our study is the sample used per group, which can be extrapolated to a broader population. However, it should be noted that despite the fact the sample is a complete high-level team, the reduced sample prevents us from assessing relevant differences in the position of the player on the court and this limits the possibility of knowing if the dominance of the lower limb in the jump can depend on the position of each player on the court.

In addition, the body composition variable has been taken during the season, and it would have been interesting to assess the changes including the preseason and postseason and the menstrual cycle should have been taken into account at the time data collection. Moreover, the limited availability of the elite team hampered the data collection process.

## Conclusions

The players presented a relevant percentage of asymmetries in the muscle power parameters during the CMJ test between the dominant and non-dominant lower limbs. In addition, differences were observed between players in jumping performance in this sample. However, despite using the main gold standard instruments in the field (e.g. DXA) and showing differences between limbs in body composition parameters, dominance did not appear to show a relevant role in the development of asymmetries. That is why these differences were associated with the development of functional asymmetries in a sport such as handball.

On the other hand, the presence of asymmetries in lean mass between the right and left leg was not shown to be relevant in the development of asymmetries in the power of the lower extremities during the CMJ in the different phases of the jump.

For this reason, it is concluded that the presence of asymmetries in handball must be assessed. However, it does not have to be one of the main parameters to be taken into account by coaches of elite athletes.

Furthermore, highlight the importance of including specific analyzes of body composition and sports performance on an individualized basis in elite players. To this end, it is recommended to include more specific training programs and focus training on eccentric exercises to reduce the risk of asymmetries between extremities. In addition, shows the importance of work plyometric exercises and unilateral actions in the different planes of movement that resemble the actions of the players on the court. The aim is assessing in more detail the dominance of the extremities during these exercises, in order to improve sports performance and avoid the risk of sports injuries.

## Data Availability

The datasets used and/or analyzed during the current study are available from the corresponding author upon reasonable request.
